# Clinical Manifestations of Zika Virus Infection, Rio de Janeiro, Brazil, 2015

**DOI:** 10.3201/eid2207.160375

**Published:** 2016-07

**Authors:** José Cerbino-Neto, Emersom Cicilini Mesquita, Thiago Moreno L. Souza, Viviane Parreira, Bernardo Bastos Wittlin, Betina Durovni, Maria Cristina Ferreira Lemos, Alexandre Vizzoni, Ana Maria Bispo de Filippis, Simone Alves Sampaio, Bianca de Santis Gonçalves, Fernando A. Bozza

**Affiliations:** Fundação Oswaldo Cruz, Rio de Janeiro, Brazil (J. Cerbino-Neto, E.C. Mesquita, T.M.L. Souza, V. Parreira, A. Vizzoni, A.M. Bispo de Filippis, S.A. Sampaio, B. de Santis Gonçalves, F.A. Bozza);; Secretaria Municipal de Saúde do Estado do Rio de Janeiro, Rio de Janeiro (B.B. Wittlin, B. Durovni, M.C.F. Lemos)

**Keywords:** Zika virus, Zika fever, symptom, surveillance, Flaviviridae, flavivirus, exanthema, headache, fever, arthralgia, myalgia, joint swelling, conjunctivitis, public health emergency of international concern, PHEIC, Rio de Janeiro, Brazil, viruses, rash

**To the editor:** Zika virus infection, which has been associated with microcephaly and other neurologic disorders, has reached the level of public health emergency of international concern (*1*). Zika virus (family *Flaviviridae*, genus *Flavivirus*) is transmitted by mosquitos of the genus *Aedes* (*2*). The virus was first isolated from a serum specimen from a rhesus monkey in the Zika Forest of Uganda in 1947 (*3*). After 2007, a rapid geographic expansion of the virus was observed, including outbreaks in the Pacific region (*4*) and, more recently, in South America. Brazil reported the first autochthonous case of Zika virus disease in April 2015 (*5*), and subsequently, increasing numbers of cases have been reported, especially in northeastern Brazil (*6*). 

Studies on the natural history of Zika virus infection are scarce. Previous research defined Zika virus infection as a dengue-like illness, typically characterized by fever, maculopapular rash, arthralgia, and conjunctivitis (*4*). Although some patients have all of these symptoms during early onset, fever is not an early symptom for all. Here we describe the frequency of signs and symptoms from a sample of clinic patients in Rio de Janeiro, Brazil, who were later confirmed to have Zika virus disease by using real-time reverse transcription PCR (rRT-PCR).

We retrospectively collected clinical data on a convenience sample of 57 patients found to be Zika virus–positive by rRT-PCR who had medical attention at the 24-hour acute care clinic of Manguinhos in Rio de Janeiro during April 28–June 8, 2015. Data were collected from electronic medical records and surveillance reports. Data were anonymized and included age, sex, and signs and symptoms documented on the first clinic visit of patients who reported acute rash, dengue-like illness, or both. Fever was documented either through direct measurement in the clinic or by patient self-report. Pregnancy status was not assessed. We collected blood samples for serum sample testing during each patient’s initial visit to the clinic and tested for Zika virus using rRT-PCR as described by Lanciotti et al. (*7*); all samples were collected within 7 days of illness onset. Patients were not tested for dengue or chikungunya viruses. We did not measure the duration of any sign or symptom.

Of the 57 Zika virus disease case-patients, median age was 34 years; 63% were women (Table). The most common sign or symptom was exanthema (98%), followed by headache (67%), fever (67%), arthralgias (58%), myalgias (49%), and joint swelling (23%) (Table). Conjunctivitis was observed in 39% case-patients and retro-orbital eye pain was reported by 40%. Among 30 patients who had fever assessed by clinic staff, median temperature was 38°C (range 37.5°C –38.5°C). One patient had no rash or joint swelling but did have all other symptoms. One patient’s sole symptom was rash. No patients were referred for hospitalization.

**Table:** Characteristics of Zika virus disease patients seeking care in an acute care clinic, Rio de Janeiro, Brazil, April 28–June 8, 2015

Characteristic	Value*
Cohort, no. patients	57
Age, y	34 (25–40)
Female sex	36 (63)
Symptoms	
Exanthema	56 (98)
Fever†	38 (67)
Days from symptom onset to exanthema	1 (0–2)
Arthralgia	33 (58)
Itching	32 (56)
Headache	38 (67)
Myalgia	28 (49)
Retro-orbital pain	23 (40)
Conjunctivitis	22 (39)
Joint swelling	13 (23)

*Median (interquartile range) or no. (%) case-patients. †Measured in medical office (n = 30) or self-reported (n = 8).

Our clinic-based study of 57 rRT-PCR–confirmed cases of Zika virus disease found rash to be the most common symptom for which patients sought care (98%); fever, generally low-grade, was reported or observed in 67%. Because our study design was retrospective in nature, wherein we reviewed records for selected patients in whom Zika was subsequently found to be laboratory-confirmed by using rRT-PCR, we may have introduced selection bias to our sample, limiting the generalizability and comparability of our results. For example, clinic staff may have seen patients with mild symptoms but decided not to test for the virus, leading to a bias toward testing patients with more severe rash. It is also possible, considering the retrospective nature of our data collection, that some data points were not accurately recorded and could not be validated. Despite these limitations, our data suggest the term “Zika fever” is not a helpful substitute term for Zika virus disease. Furthermore, referring to the illness caused by this virus as “Zika fever” (*8*) may be misleading and should probably be avoided until further more systematic studies clarify the frequency of fever as a symptom.

Although patient sampling and laboratory testing methods are not directly comparable to our study, a 2015–2016 assessment in Puerto Rico detected Zika virus in 30 of 155 case-patients in whom Zika virus disease was suspected. In that study, laboratory-confirmed disease was defined as detection of Zika virus RNA by using rRT-PCR or IgM by using ELISA. Among the 30 confirmed cases, the most frequently reported signs and symptoms were rash (77%), myalgia (77%), arthralgia (73%), and fever (73%) (*9*). The February 12, 2015, interim case definition published by the World Health Organization describes a suspected case-patient as a person with rash, fever, or both, in addition to 1 of 3 other listed symptoms (*10*). Like the Puerto Rico report, our report supports the established World Health Organization case definition indicating that the presence of rash, fever, or both should be emphasized as primary characteristics of Zika virus disease.
